# Meditation programs for adults with type 2 diabetes mellitus: A protocol for a systematic review and meta-analysis

**DOI:** 10.1097/MD.0000000000031459

**Published:** 2022-11-25

**Authors:** Jiayu Xu, Manxue Mei, Haoxiang Sun, Xiaofang Chen, Wei Zhu, Jianping Song

**Affiliations:** a Artemisinin Research Center, Guangzhou University of Chinese Medicine, Guangzhou, China; b The Second Clinical College, Guangzhou University of Chinese Medicine, Guangzhou, China.

**Keywords:** meditation programs, protocol, systematic review, type 2 diabetes mellitus glycosylated hemoglobin fasting blood glucose

## Abstract

Type 2 diabetes mellitus is intimately linked to chronic stress. Meditation programs belong to mind-body therapies, which could benefit patients’ disease management. Though some clinical trials have proved that meditation programs have the ability to improve level of blood glucose quality of life, body mass index and blood indexes related to metabolism in individuals with type 2 diabetes mellitus, the efficacy of meditation programs needs further confirmation. Thus we will conduct this systematic evaluation and meta-analysis to summarize and analyze all the results included to obtain reliable evidence.

**Methods:**

We will search several English and Chinese databases for relevant clinical trials published up to July 2021, and randomized controlled trials or controlled trials among adults with type 2 diabetes mellitus are included. Two reviewers will extract data and assess the quality of included studies independently. The main outcomes of this research are glycosylated hemoglobin level and fasting blood glucose level. The secondary outcomes are high-density lipoprotein, low-density lipoprotein, body mass index, remission of depression and anxiety, and quality of life. Stata v.14.0 and Review Manager V5.3 will be used to synthesize and analyze all data included.

**Results:**

Grading of Recommendations Assessment, Development, and Evaluation will be used to evaluate the quality of the assessments. Our study will be disseminated through publications in peer-reviewed journals.

**Conclusion:**

This systematic review is the first to analyze the efficacy of different types of meditation for type 2 diabetes mellitus, which could provide evidence for the use of mediation programs as non-drug approaches.

**Trial registration number:**

PROSPERO CRD42021274508.

## 1. Introduction

According to the International Diabetes Federation Diabetes Atlas (10th edition) published by World Health Organization, nearly 537 million adults suffer from diabetes mellitus (DM) globally, and this number is projected to reach 643 million by 2030, and 783 million by 2045,^[1]^ and type 2 diabetes (T2DM) with a feature of insulin resistance is the most common form of diabetes.^[2]^ T2DM could trigger numerous serious complications, including microvascular, macrovascular complications and diabetic neuropathies,^[3,4]^ and increases the risk of stroke, cognitive impairment and cancer.^[5–7]^ A survey from International Diabetes Federation indicates that the global economic burden of diabetes-related diseases is around one trillion USD and will exceed this figure by 2030.^[1]^

The development of T2DM is closely associated with low-level, chronic inflammation, insulin resistance and various metabolic disorders.^[8–10]^ Human lifestyle alterations, like prolonged sedentary lifestyle, depression and anxiety, intake of refined sugar and high-fat diet, have contributed to a rapid increase of T2DM worldwide.^[11,12]^ Demand of modern life makes people suffer from chronic stress for a long time.^[13]^ Chronic stress could cause excitement of sympathetic nerve, leading to activation of the hypothalamus-pituitary-adrenal gland (HPA).^[14]^ The activation of HPA could trigger the release of glucocorticoids and cortisol, affecting the body’s blood sugar homeostasis, which could finally cause insulin resistance.^[15]^ Additionally, there is a currently considerable evidence of an association between negative stress (like depression and anxiety) and T2DM. DM is reciprocally linked to depression through many ways.^[16]^ Researches have proved that depression may make the body in a low-grade inflammation state, and C-reactive protein, as an inflammatory-related factors, partially mediates the relationship between depression and diabetes.^[17]^ Negative emotional state could be changed by mind-body therapies. As a non-drug approach for T2DM, mind-body therapies have gained more attention than ever before, which could also help reduce social medical costs.

Meditation is a kind of mind-body interventions, a form of mental training that helps individuals train their attention and awareness to achieve mental well-being.^[18]^ Derived from religion, meditation has many forms, like transcendental meditation (TM) and mindfulness meditation.^[19]^ It could also be combined with some physical exercises to forms of yoga and Tai Chi. At present, meditation programs have been widely used to treat depression, anxiety, insomnia and other psychological problems.^[20–22]^ Additionally, meditation may also benefit to other stress-related medical diseases, such as chronic obstructive pulmonary disease, hypertension, and chronic pain.^[23–25]^ In 2013, the American Heart Association has recommended transcendental meditation as an alternative therapy for hypertension with level IIb evidence.^[26]^ Meditation could reduce many indicators related to the metabolic syndrome, such as blood pressure, blood lipid levels, blood sugar level, glycosylated hemoglobin and body mass index (BMI).^[27]^ Clinical trials have found that meditation programs could also affect HbA1c level and improve quality of life of T2DM patients.^[28,29]^

Though the mechanism of meditation for T2DM is not clear enough,^[25]^ some studies have found that meditation projects may improve the symptoms of T2DM patients through many ways. The development and progression of T2DM is associated with the dysfunction of HPA axis, which could be regulated by meditation.^[15]^ A study found that emerging adults trained with transcendental meditation have lower morning cortisol level and cortisol awakening response.^[30]^ Inflammation plays an important role in T2DM. A meta-analysis in 2017 has summarized the influence of meditation for T2DM, and the results show that mindfulness meditation could reduce the activity of nuclear factor kappa-light-chain-enhancer of activated B cells, lower the level of C-reactive protein and increase the level of CD4+.^[31]^ Furthermore, some researches have confirmed that meditation programs have positive impacts on the length of telomeres and reduce the reactive oxygen species, and reduce the development of DM related to aging.^[32]^

Now, meditation has received more attention among people. There are also a number of clinical trials exploring the impact of various meditation programs on T2DM patients and gained considerable results.^[25,33]^ But discrepancies also exist in these studies and there is a lack of lateral contrasts among researches related to different meditation programs. Therefore, this meta-analysis aims to analyze the random clinical trials (RCTs) and controlled trials studies about meditation programs, further evaluate the effectiveness and safety of meditations program for T2DM, and find out whether there are any differences in the efficacy of different meditation programs for T2DM.

## 2. Methods

### 2.1. Study design

This protocol follows preferred reporting items for systematic review and meta-analysis protocols (PRISMA-P) guideline.^[34]^And this study will be performed in agreement with PRISMA guidelines.^[35]^

### 2.2. Registration

The protocol for the systematic review was registered with the International Platform of Registered Systematic Review and Meta-analysis Protocols (no. 2021100008).

### 2.3. Eligibility criteria

#### 2.3.1. Participants.

Adults diagnosed with T2DM are included in this study. In this review, the definition for T2DM should follow relevant guidelines, such as American Diabetes Association standard for clinical diagnosis and treatment of diabetes.

#### 2.3.2. Interventions.

Our protocol contains researches taking different forms of meditation as treatment, whether as a primary or complementary therapy. It also includes some other comprehensive therapies including meditation, such as yoga and Tai Chi.

#### 2.3.3. Controls.

Placebo treatment, treatment as usual or no treatment.

#### 2.3.4. Outcomes.

The main outcomes will be fasting blood glucose levels and glycosylated hemoglobin. The second outcomes are remission of depression and anxiety level, quality of life, BMI, high-density lipoprotein cholesterol, low-density lipoprotein and level of blood pressure.

#### 2.3.5. Study types.

This study includes RCTs, controlled trials, while case studies, conference papers, reviews, comments, retrospective cohort studies, cluster trials, cross-sectional studies are excluded.

### 2.4. Search strategy

We will search literature databases including Embase, PubMed, MEDLINE, Cochrane Library, Ovid database, China National Knowledge Infrastructure, Wanfang and VIP information databases. And then all databases mentioned above will be retrieved from 1990 to present. Details of how PubMed will be retrieved are provided in Table 1.

**Table 1:** Search strategy (PubMed).

Search	Query
1#	“meditation” [MeSH Terms]
2#	“meditate”[Title/Abstract] OR “meditated”[Title/Abstract] OR “meditating”[Title/Abstract] OR “meditation”[Title/Abstract] OR “meditations”[Title/Abstract] OR “meditational”[Title/Abstract] OR “meditative”[Title/Abstract] OR “meditator”[Title/Abstract] OR “meditators”[Title/Abstract] OR “transcendental”[Title/Abstract] OR “transcendental meditation”[Title/Abstract] OR “minds”[Title/Abstract] OR “minded”[Title/Abstract] OR “mindful”[Title/Abstract] OR “mindfulness”[MeSH Terms] OR “minding”[Title/Abstract] OR “meditated”[Title/Abstract] OR “meditating”[Title/Abstract] OR “meditative”[Title/Abstract] OR (“yoga”[MeSH Terms] OR “tai ji”[Title/Abstract] OR “tai ji”[Title/Abstract] OR “Tai Chi”[Title/Abstract]) OR “yoga”[Title/Abstract]) OR “Yogic”[Title/Abstract]
3#	“Diabetes Mellitus, Type 2” [MeSH Terms]
4#	“Type 2 Diabetes Mellitus” [Title/Abstract] OR “Type 2 Diabetes” [Title/Abstract] OR “T2DM” [Title/Abstract] OR “Diabetes, Type 2” [Title/Abstract] OR “Diabetes Mellitus, Type II” [Title/Abstract] OR “Insulin resistance” [Title/Abstract] OR “NIDDM” [Title/Abstract] OR “Stable Diabetes Mellitus” [Title/Abstract] OR “Diabetes Mellitus, Noninsulin-Dependent” [Title/Abstract] OR “Adult-Onset Diabetes Mellitus” [Title/Abstract] OR “Diabetes Mellitus, Type II” [Title/Abstract] OR “Non-Insulin-Dependent Diabetes Mellitus” [Title/Abstract] OR “Diabetes Mellitus, Maturity-Onset” [Title/Abstract]
6#	1# OR 2#
7#	3# OR 4#
8#	5# AND 6#

### 2.5. Study selection and data extraction

#### 2.5.1. Study selection.

**Figure 1.**
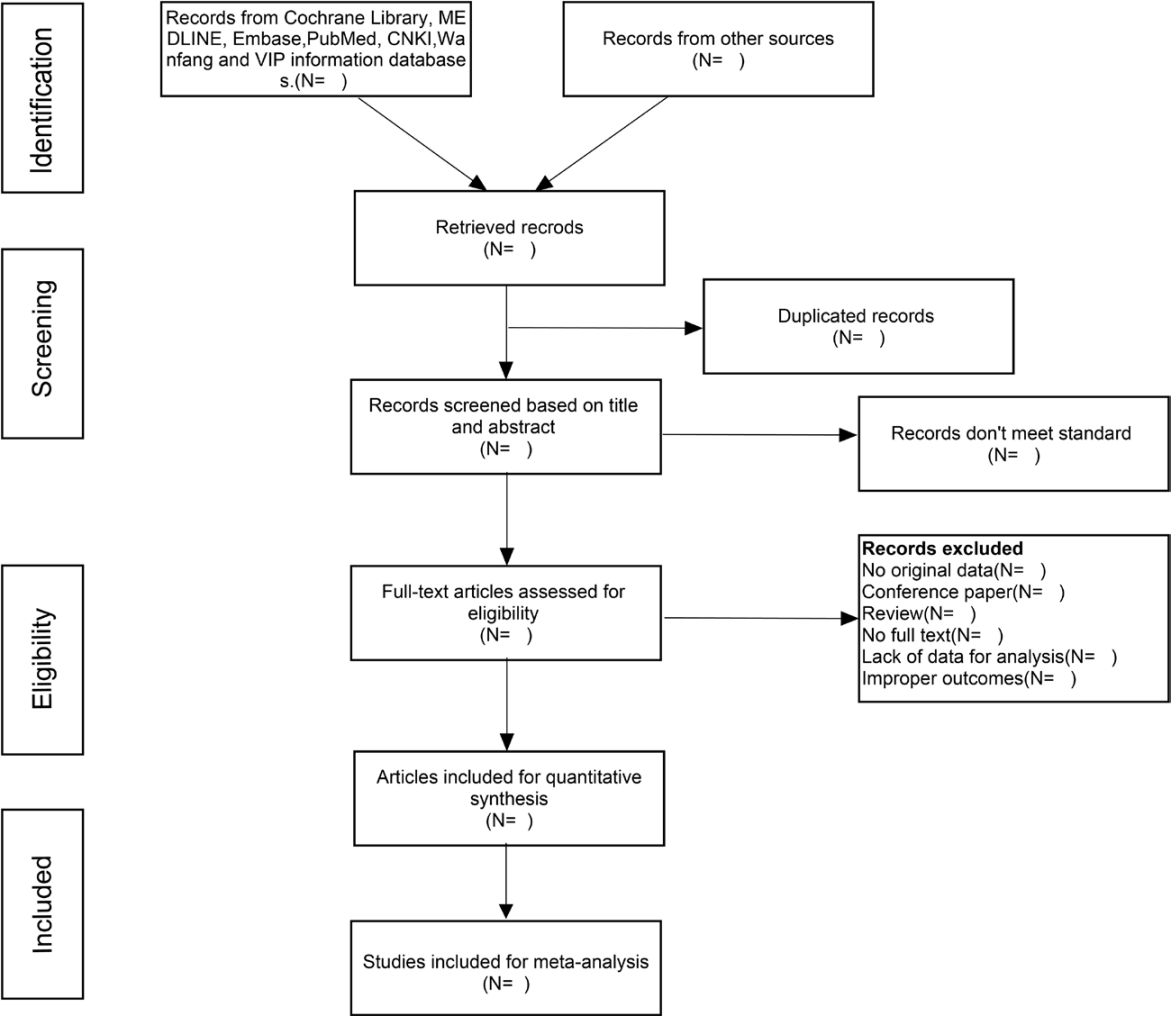
Flow chart and descriptions of study selection.

Two review authors will scan all literatures’ titles, abstracts, and full text according to eligibility criteria, and remove repeated and irrelevant studies. If there is any doubt about studies screened in this process, the articles will be evaluated again by a third reviewers besides them. Researches will be retrieved further which met the inclusion criteria. Figure 1 presents PRISMA flow diagram of the study selection process.

#### 2.5.2. Data extraction.

Two reviewers will extract data independently according to a standard model we made before. Any dissents between them will be solved with assistance of a third reviewer. Specific extraction contents are as follows: essential information of included studies (first author, country, year of publication), design of study (randomization, blinding), details of participants (age, gender, duration of T2DM, total number of participants), intervention and control (type of meditation, duration of treatment), and outcome measurements (HbA1c, fasting blood glucose levels, remission of depression and anxiety level, quality of life, BMI, and level of blood pressure). Data not provided in published articles will be collected from corresponding authors by e-mail.

### 2.6. Quality assessment of included studies

Cochrane Risk of Bias tool will be used to assess random sequence generation, allocation concealment, blinding of participants and personnel, blinding of outcome assessment, incomplete outcome data, selective reporting and other bias of all included studies. Then make judgment for every study with low risk, unclear risk, and high risk. And Newcastle-Ottawa-Scale will be used to evaluate studies which are not RCTs.

### 2.7. Data synthesis and statistical analysis

All statistical analyses will be performed using Revman5.3.0 (Cochrane information management system) and Stata V.16.0 software. For continuous outcomes, we will calculate mean differences with 95% CIs and *I*^2^ statistic for heterogeneity across studies. A value of *P* < .05 would be considered statistically significant. If there was homogeneity among studies (*P* > .1, *I*^2^ < 50%), fixed-effect model will be used. Random effect model would be used to analyze when there was heterogeneity among the studies (*P* ≤ .1, *I*^2^ > 50%).

### 2.8. Sensitivity and subgroup analyses

We will also perform a sensitivity analysis by excluding studies with high risk of bias. Subgroup meta-analyses and meta-regressions are conducted to explore reasons of heterogeneity, such as types of study design, populations and different meditation programs.

### 2.9. Assessment of publication bias

Egger test will be conducted to evaluate publication bias, and results displayed by funnel plot. If there is publication bias (*P* < .05), we would use a trim and fill method to adjust bias.

### 2.10. Quality assessment

Two authors will independently assess included trials’ methodological with the Grading of Recommendations Assessment, Development, and Evaluation guidelines (GRADE).^[36]^

### 2.11. Ethics and dissemination

This study is a meta-analytic review without patients enrolled, therefore ethics approval is not essential. After finish this study, all results will be submitted to peer-reviewed journals.

## 3. Discussion

Previous systematic reviews have confirmed that mind-body interventions could affect serum glucose levels, quality of life of patients with T2DM. So far, our study is the first to analyze effects of different meditation projects for type 2 diabetes, and would provide a detailed summary of available evidence. This review may be limited by the quality of all included studies. The potential risk of bias in each study would also be assessed. T2DM patients are always with metabolic disorders, so we would further assess the impact of meditation programs on metabolic related indicators, like low-density lipoprotein, high-density lipoprotein cholesterol, and BMI. Studies have shown that the incidence of depression is higher in individuals withT2DM, and this protocol would evaluate the improvement of depression and anxiety of patients. Our protocol provides a clear and structured procedure to extract relevant information comprehensively and provide the effects of meditation projects on patients with T2DM and compared the differences among meditation programs. The results of this systematic review could be a interest for patients and physicians who pay attention to mind-body interventions and provide evidence for it to be included in the guidelines as a part of non-drug approaches.

## Acknowledgments

We appreciate the support of the fund and all authors who participated.

## Author contributions

JX and JS designed the study. JX, MM, and WZ drafted and registered the protocol. JX, MM, and HS will screen the studies for data analysis. JS, WZ, JX, and MM revised the protocol. All authors read and approved the final manuscript.

**Data curation:** Jiayu Xu, Xiaofang Chen.

**Formal analysis:** Jiayu Xu, Xiaofang Chen.

**Funding acquisition:** Manxue Mei, Jianping Song.

**Investigation:** Manxue Mei.

**Methodology:** Haoxiang Sun.

**Resources:** Manxue Mei, Haoxiang Sun.

**Visualization:** Jiayu Xu.

**Writing – original draft:** Jiayu Xu.

**Writing – review & editing:** Jiayu Xu, Wei Zhu, Jianping Song.
